# Digital health citizens and the future of the NHS

**DOI:** 10.1177/2055207616672033

**Published:** 2016-10-10

**Authors:** John Powell, Nikki Newhouse, Anne-Marie Boylan, Veronika Williams

**Affiliations:** University of Oxford, Oxford, UK

Health systems worldwide are facing unprecedented challenges as they seek to deliver better value healthcare against a backdrop of increasing levels of chronic disease, ageing populations, global financial crises and reduced public spending, and digital health tools and services are widely touted as being part of the answer, offering low-cost and patient-centred solutions.^1^ The notion of a digitally sophisticated health service user, situated at the heart of the health service, is one which catches the public and commercial imagination but is yet to be realised. To achieve this ‘citizen health’ vision of a technology-enabled upstream health system centred around users who are provided with the tools and opportunities to be active participants in the consumption and production of their health and health services, the digital health citizen needs to take on new responsibilities, alongside their digital ‘rights’, and health services need to support and harness these. At the same time, it is obviously important to be mindful of issues of digital exclusion, where there are potential issues of unequal access, unequal use and unequal outcomes for people using digital tools.

In England, the National Health Service (NHS) has stated a clear commitment to a digital future,^2^ but in order to achieve its aims the current model of service provision – in the NHS and elsewhere – needs fundamental transformation. Health services are perhaps better characterised as sickness services designed around expensive secondary care facilities. A genuine focus on prevention and well-being promotion is likely to be far more effective in reducing costs downstream. But the prioritisation and maintenance of a preventive focus is hindered by the frequency of the political election cycle and the need for health services to demonstrate short-term benefits. To be sustainable, state-funded health systems must find innovative ways to engage citizens in their own care both when ill and, critically, when healthy. The leading US physician and patient safety expert Professor Bob Wachter has recently led a review to examine the implementation of digital technology in the NHS, but in the terms of reference of this review ‘patients’ were only mentioned once, in respect of their relationship with clinicians. Healthy citizens were not mentioned at all.^3^ Yet, digital health offers a way to not only bridge the gap between professional care and self-management, but also to engage people in changing health behaviours.

In an influential 2014 article in the Harvard Business Review, Jeremy Heimans and Henry Timms argued that new digital technologies are causing a shift in the business sector from a model of ‘old power’ which is principally a consumption model grounded in what a few individuals or organisations know, own or control, to a model of ‘new power’ which is characterised by a participatory approach of peer coordination and harnessing the agency of the crowd.^4^ If the business of healthcare is to harness the benefits of new power, to be truly patient-centred and participatory, then it is vital that we embrace open debate about the rights, responsibilities and expectations of future digital health citizens. This debate needs to happen not only amongst healthcare professionals and patients within the NHS, but also within society as a whole.

Digital technologies are ubiquitous and readily utilised by the majority of citizens. The idea of taking responsibility for our affairs digitally is no longer alien to us: banking, travel bookings, shopping and general communication are now all routinely conducted online. In healthcare, digital tools can improve efficiency, effectiveness and safety, and enable people to become more engaged in their own care. But technology on its own will not drive health service change, and the simple provision of technology at the point of care does not equate to engagement. Change needs to be driven by people: by policymakers, health professionals, patients and, importantly, from citizens. As a national service funded through general taxation, it can be argued that preserving and improving the NHS is a responsibility for every citizen. As stated by the Department of Health, ‘the NHS belongs to us all’.^5^ The social contract that the NHS has with its users is embodied in the NHS Constitution, but only 24% of the British public were aware of its existence.^6^ It outlines the rights and responsibilities of both staff and patients, and the NHS at large. Patients and the public have rights to have access to data and information, and the responsibility to provide feedback, but no specific rights or responsibilities in relation to digital technologies. We believe there is a need for a new social contract between a digital NHS and its users, in order to encourage a cultural shift that allows digital tools to enable and empower us in our roles as proactive participants in our own well-being. If we want to create this digital future, indeed if we want to co-create it, then the rights and responsibilities of the *digital health citizen* of the 21st century need to be established. In Box 1 we have presented some initial proposals to start this debate.

**Box 1.** Potential rights and responsibilities of a digital health citizen.

**Rights of the digital health citizen**
• To have a health system which makes best use of digital technologies to keep them healthy and is able to intervene early when required.
• To be provided with a usable, efficient personal health record (PHR) which has obvious benefits to them.
• To be provided with the digital tools to support a healthy lifestyle.
• To be provided with the digital tools to support remote management and self-management of health conditions.
• To have a health system which shares data responsibly to support coordinated care.
• To have a health system which uses its data, including feedback from users, to learn and improve.
• To know what happens to their data.
• To have a health service which harnesses information technology to ensure patients never experience a ‘never event’.
• To be supported in the use of digital tools, through the provision of appropriate training or other resources to themselves or their carers.
**Responsibilities of the digital health citizen**
• To maintain an up-to-date and accurate digital PHR.
• To share their PHR data with their healthcare provider.
• To share their PHR data with researchers and health service planners and policymakers.
• To contribute lifestyle data to their PHR.
• To use digital tools including apps to maximise their health and well-being.
• To participate in remote consultations.
• To use wearable technologies to facilitate the remote monitoring of their health status.
• To share their experiences of health and illness and to provide online feedback on all care interactions.
• To accept some of the administrative burden of health data management.

Establishing a patient-centred culture of digital innovation and improvement could lead to an NHS that better serves its users. Engagement could include using a personal health record which shares data with the NHS and health service researchers, contributing data from wearable technologies that monitor parameters of health or illness, using mobile apps that support decision making or deliver interventions, participating in remote care and consultations, and providing online ratings and reviews of experiences of care. Digital health citizenship could play a crucial role in ensuring that patients and the public are firmly placed at the heart of the health service, a popular message that is not yet the reality of healthcare practice.
